# Effectiveness of Tranexamic Acid in Trauma Patients: A Systematic Review

**DOI:** 10.7759/cureus.52111

**Published:** 2024-01-11

**Authors:** Kenneth Meza Monge, Sabrina S Domene, Diana L Diaz Mendoza, Andrea Vidal-Gallardo, Adriana M Alfaro Llique, Miguel Rodriguez, Pooja Premchandra, Samira Anwar Pandya, Victor S Arruarana, Kenneth Aleman Paredes, Ernesto Calderon Martinez

**Affiliations:** 1 General Practice, Universidad Nacional Autónoma de Mexico, Mexico City, MEX; 2 General Practice, Universidad Nacional de Mar del Plata, Mar del Plata, ARG; 3 Internal Medicine, Universidad Nacional Autónoma de Mexico, Mexico City, MEX; 4 Surgery, Universidad de los Andes, Merida, VEN; 5 General Practice, Universidad Ricardo Palma, Lima, PER; 6 Surgery, Mercury Clinical Research, Houston, USA; 7 General Practice, American International Medical University, Gros Islet, LCA; 8 General Practice, Kenyatta National Hospital, Nairobi, KEN; 9 Internal Medicine, Brookdale University Hospital Medical Center, New York, USA; 10 Surgery, Hospital General Regional No. 220 "Jose Vicente Villada", Toluca, MEX; 11 Digital Health, Universidad Nacional Autónoma de Mexico, Mexico City, MEX

**Keywords:** coagulopathy, intraoperatively, preoperatively, intravenous administration, emergency surgery, trauma patients, hemostatic agent, tranexamic acid

## Abstract

Tranexamic acid (TXA), a fibrinolytic agent, effectively inhibits plasminogen activation, thereby reducing fibrinolysis and hemorrhage. This study focused on its application in trauma patients undergoing emergency surgery, a critical area due to trauma's significant role in mortality. Our investigation involved a meticulous screening of randomized controlled trials from databases including Scopus, PubMed, Web of Science, and Cochrane. The findings indicate that TXA intervention is promising in enhancing outcomes for trauma patients. However, the drug's effectiveness may vary based on the specific nature of the medical condition. In summary, robust evidence suggests that TXA can diminish blood loss, lower transfusion rates, reduce complications, and improve hemoglobin and hematocrit levels in surgical patients. Consequently, TXA should be considered a crucial medication, readily available to mitigate morbidity and mortality in surgical settings. Future research should explore factors influencing TXA's effectiveness in traumatic brain injury cases and across a broad spectrum of surgical scenarios in diverse patient populations. This would further guide clinicians in refining and optimizing the use of TXA.

## Introduction and background

Tranexamic acid (TXA), an antifibrinolytic agent, plays a pivotal role in preventing exsanguination by competitively inhibiting plasminogen activation, thereby reducing fibrinolysis and hemorrhage [1]. Initially utilized in patients with bleeding disorders, TXA was found to be applicable in managing hemophiliacs undergoing oral surgical interventions and in treating menorrhagia [2]. Over time, TXA's versatility has been harnessed to prevent bleeding complications in various medical and surgical scenarios, including traumatic injury and postpartum hemorrhage [3]. Trauma, the leading cause of mortality in individuals aged one to 44 years in 2017 [4], underscores the critical need for effective interventions. Large randomized controlled trials (RCTs) consistently highlight TXA's survival advantage in acute bleeding scenarios without elevating thromboembolic risks [1]. As a valuable tool in trauma surgery, TXA demonstrates the potential to curtail blood loss and reduce transfusion requirements [5]. Its capacity to bolster clot formation and alleviate trauma-induced coagulopathy positions TXA as a promising intervention for enhancing outcomes in trauma patients [6].

While extant research underscores the potential benefits of TXA, discrepancies exist, with some studies presenting conflicting findings regarding its efficacy in specific subsets of trauma patients, one study conducted a systematic review and bias-adjusted meta-analysis of RCTs, highlighting the significant reduction in the risk of death due to bleeding with TXA, without an increased risk of vascular occlusive events [7]. Conversely, another study emphasized the ongoing investigation into the optimal dosing and timing of TXA administration in non-trauma surgical populations, indicating the lack of conclusive evidence in this patient group [8]. These findings echo those of other reports in the literature, suggesting that the debate on TXA’s benefits in trauma injury surgeries remains unresolved [9,10]. These divergences emphasize the imperative for a comprehensive systematic review to amalgamate available evidence, address methodological variations, and offer a nuanced understanding of TXA's overall impact on this patient population. Such a review is indispensable for informing clinical practice, facilitating evidence-based decision-making, and pinpointing areas for future research to optimize TXA use in trauma patients undergoing emergency surgery.

## Review

Methods

This study conducted an extensive systematic review following the 2020 guidelines outlined in the Preferred Reporting Items for Systematic Reviews and Meta-Analyses (PRISMA) [11,12].

Criteria for Considering Studies in This Review

Types of studies: For our research, we conducted a systematic review of relevant studies published from 2000 to 2023, available in English. We meticulously screened RCTs and semi-RCTs. These study designs are chosen to ensure a rigorous evaluation of the effectiveness of TXA in trauma patients undergoing emergency surgery. We excluded systematic reviews, case reports, case series, dissertations, book chapters, protocol articles, reviews, news articles, cohorts, cross-sectional studies, conference abstracts, letters to the editor, editorials, and comment publications. Furthermore, we excluded studies that did not clearly describe their operationalization, duplicates, and those for which we could not obtain the necessary data, see or look at the article, or receive a response from the original author via email.

Types of participants: This study has set specific participant selection criteria, including adult patients who are over 18 years old including both genders, with a high focus on trauma patients of all age groups, encompassing both adults and the elderly who undergo emergency surgery due to traumatic injuries. There are no restrictions based on the severity of trauma or specific types of traumatic injuries. We excluded studies involving pediatric populations (under 18 years of age), animals, models, or in vitro studies.

Types of intervention: To be eligible for inclusion in this study, the selected research must compare and evaluate TXA administration as a hemostatic agent, preoperatively or intraoperatively, during emergency surgery for trauma patients. The focus is on evaluating the impact of TXA on various outcomes related to blood loss and hemostasis in the context of emergency surgical interventions. The control group can receive no intervention, standard care, or alternative intervention. We excluded studies that do not involve the current intervention.

Outcomes: The primary outcome of interest is reduced blood loss during and after emergency surgery. Secondary outcomes include transfusion requirements, the incidence of reoperation due to bleeding, mortality rates, and the incidence of thromboembolic events. These outcomes are selected to comprehensively assess the effectiveness and safety of TXA in the specified population.

Searching Methods

Inclusion and exclusion criteria were used to select only high-quality studies for analysis. A rigorous exclusion criterion was applied to ensure the quality and relevance of the studies included in the analysis. We conducted a search on 11/11/2023 on Scopus (Table 1), PubMed (Table 2), Web of Science (Table 3), and Cochrane (Table 4), using Medical Subject Heading (MeSH) terms and free text terms.

**Table 1:** Specific search for Scopus database

Search	Results
TITLE-ABS-KEY (tranexamic AND acid AND trauma AND emergency AND surgery)	147

**Table 2:** Specific search for PubMed database MeSH: Medical Subject Heading.

Search	Results
(((Tranexamic Acid[MeSH Terms]) OR (Tranexamic Acid[Title/Abstract]) OR (Tranexamic[Title/Abstract])) AND ((Trauma[MeSH Terms]) OR (Trauma[Title/Abstract]) OR (accident[Title/Abstract]) OR (trauma*[Title/Abstract])) AND ((Emergency Surgery[MeSH Terms]) OR (Emergency surgery[Title/Abstract]) OR (Surgery of emergency [Title/Abstract]) OR (Rapid surgery[Title/Abstract]) OR (Surgery[Title/Abstract]) OR (Emergen* Surge*[Title/Abstract])))	434

**Table 3:** Specific search for Web of Science database

Search	Results
#1 ALL = (tranexamic acid)	8,536
#2 ALL = (Trauma)	431,224
#3 ALL = (emergency surgery)	123,784
#1 AND #2 AND #3	285

**Table 4:** Specific search for Cochrane database MeSH: Medical Subject Heading.

Line	Search	Results
1	MeSH descriptor: [Tranexamic Acid] explode all trees	1587
2	(Tranexamic acid):ti,ab,kw	3943
3	(Tranexamic):ti,ab,kw	3947
4	MeSH descriptor: [Wounds and Injuries] explode all trees	35269
5	(Trauma):ti,ab,kw	19379
6	(Accident):ti,ab,kw	20296
7	(Trauma*):ti,ab,kw	33085
8	(Emergency surgery):ti,ab,kw	5126
9	(Surgery of emergency):ti,ab,kw	5076
10	(Rapid surgery):ti,ab,kw	5284
11	(Emergen* Surge*):ti,ab,kw	9084
12	#1 OR #2 OR #3	3947
13	#4 OR #5 OR #6 OR #7	79912
14	#8 OR #9 OR #10 OR #11	13836
15	#12 AND #13 AND #14	37

Data Extraction and Selection of Studies

A screening based on titles and abstracts was conducted in the initial phase. Two independent reviewers (SSD and AMAL) then selected trials for inclusion in this review using predetermined inclusion and exclusion criteria. This process involved using Rayyan, a tool for extracting relevant data and filtering duplicates. Keywords were specifically employed to highlight inclusion and exclusion criteria-related words on Rayyan [12,13].

In disagreements regarding the inclusion of studies, consensus was reached after consultation with a third review author (ECM). Following this, a comprehensive full-text analysis was conducted. Again, the two reviewers (SSD and AMAL) independently evaluated the trials for inclusion based on the same predetermined criteria. Disagreements were similarly resolved through consensus and consultation with the third review author (ECM).

Data Evaluation: Assessment of Risk of Bias in Included Studies

The data were evaluated according to the criteria outlined in the Cochrane Handbook. To assess the quality of the included studies in this systematic review, we applied the Cochrane Risk of Bias (RoB) 2.0 tool, specifically designed for RCTs [14]. Two independent reviewers assessed the risk of bias in each study, adhering to the specific criteria and guidelines of the Cochrane Handbook. Discrepancies between the reviewers were resolved through discussion or consultation with a third, blinded reviewer.

The methodological components of the trials were evaluated for their risk of bias, categorized as low, high, or unclear, in accordance with the guidelines from the Cochrane Handbook for Systematic Reviews of Interventions. Any adjustments made to the quality of evidence, whether downgrading or upgrading, will be detailed in the summary of findings table, ensuring transparency and providing explanations for each bias assessment in the included studies.

Results

**Figure 1:**
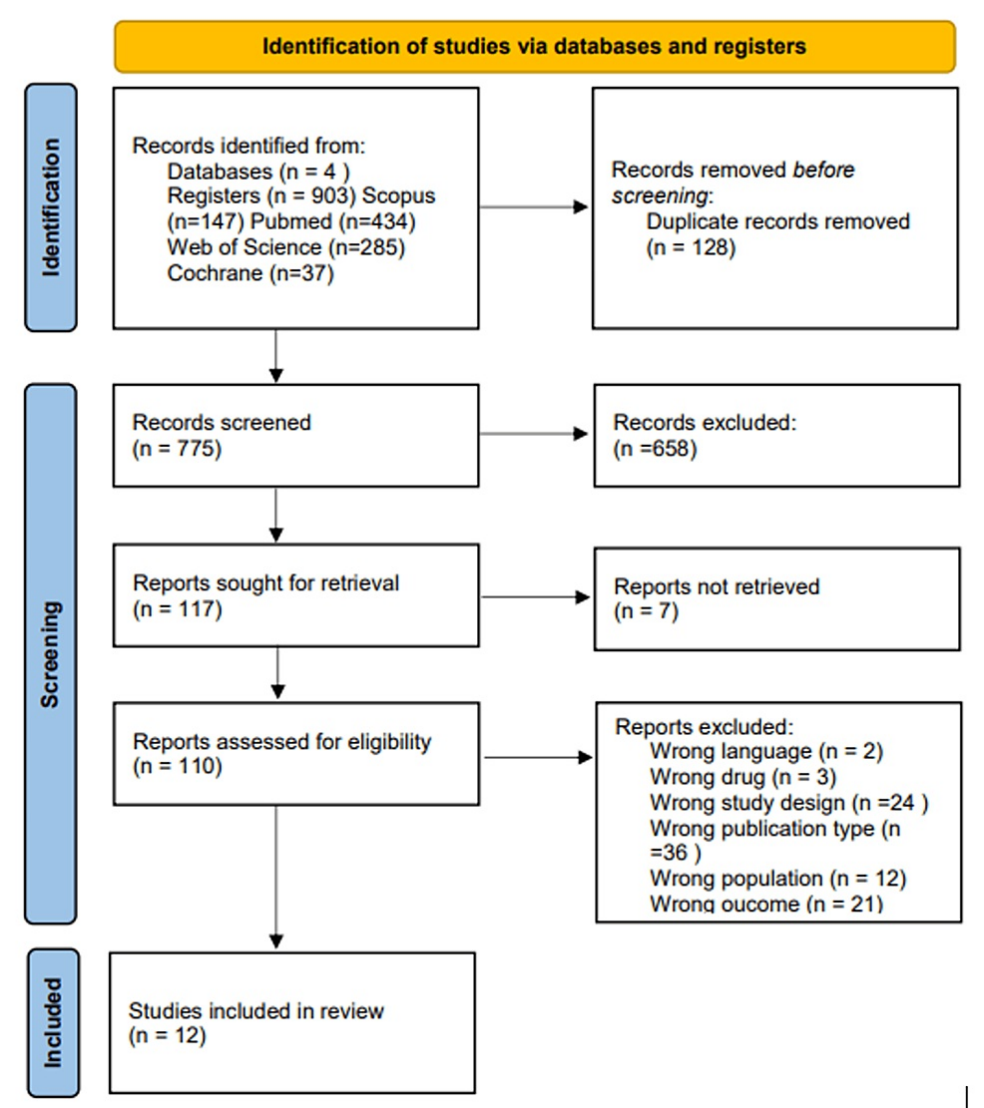
PRISMA flow diagram PRISMA: Preferred Reporting Items for Systematic Reviews and Meta-Analyses.

From the searched databases, a total of 1810 potential articles were identified. After an extensive review, 128 were found to be duplicates and subsequently eliminated. Screening of titles and abstracts led to the selection of 110 publications for further review and full-text retrieval. A meticulous evaluation of these narrowed-down articles for eligibility and quality resulted in the selection of 12 for the review process. The PRISMA flow chart illustrates the study selection procedure (Figure 1).

In this study, we analyzed data from a total of 78,188 patients from various countries to understand the impact of TXA in emergency surgery settings for trauma. The primary objective was to assess the change in patient outcomes following the administration of TXA during emergency surgery for trauma. The most prevalent type of injury among the patients was blunt trauma and bleeding found in six of the studies. This was followed by head trauma and intracranial hemorrhage in three studies. Other types of trauma observed included injuries to the lower limb and pelvis. This showed us a wide range of trauma pathologies included in our systematic review where TXA has been studied.

Regarding treatment, the most commonly administered TXA dose was 1 gram at presentation, followed by another eight hours later, as noted in four of the articles reviewed. Additionally, five of the studies administered a single dose of 1 gram of TXA upon arrival, and one study used 1 gram at presentation, followed by another dose three hours later. This implies that there is no general consensus about a standard dose and a need to verify for adverse events and previously reported doses used in different scenarios.

The findings were significant in seven studies. Administering TXA within the first hour significantly reduced the mortality rate within the first 30 days of hospital admission and led to favorable outcomes six months later. For patients with lower limb trauma, the drop in hemoglobin levels post surgery was less pronounced compared to the placebo group, as reported in one study. Moreover, in cases of pelvic trauma, TXA administration showed a significant difference in hemoglobin and hematocrit levels at 24, 48, and 72 hours compared to placebo. Early treatment with TXA in patients with mild to moderate traumatic brain injury reduced the risk of death. However, one of the studies indicated no reduction in the risk of hemorrhage or intraparenchymal expansion due to head trauma with the use of TXA compared to placebo. The results show a promising future for the use of TXA in trauma patients such as blunt, limb, or general trauma. These results are not as promising in head trauma, requiring further research on this topic. All this information is comprehensively summarized in Table 5.

**Table 5:** General outcomes of the included studies TXA: tranexamic acid; RCT: randomized controlled trial; Hb: hemoglobin; HCT: hematocrit; IV: intravenous; SATS: oxygen saturation; ED: emergency department; TBI: traumatic brain injury; CT: computed tomography scan; GCS: Glasgow Coma Scale.

Author and year	Location	Sample size	Type of population	Study design	Administration of TXA with the dose	Type of trauma	Results	General comment
Kaur et al. [15]	India	100 patients	Adults aged 18-60 years	RCT	1 g of TXA intravenously preoperatively	Lower limb trauma	Fall in Hb levels in both groups, less pronounced in the TXA group	TXA is safe; no increased risk, evaluated by clinical monitoring of side effects and patients' renal function tests
Meretoja et al. [16]	Multinational	100 patients	18 years or older	RCT	1 g of intravenous tranexamic acid	Acute intracerebral hemorrhage	No significant reduction in intracerebral hemorrhage growth within 4.5 hours of symptom onset	TXA is not useful for intracerebral hemorrhage
Monsef Kasmaei et al. [17]	Iran	106 patients	18 to 60 years, within three hours post-trauma	RCT	1 g intravenous TXA	Pelvic trauma	Significant difference in Hb and HCT levels at 24 hours, 48 hours, and 72 hours post-admission	Difference in Hb and HCT; no difference in blood pressure
Perel et al. [18]	Multinational	20,127 patients	Trauma patients with significant bleeding risk	RCT	1 g intravenous TXA	General trauma	TXA reduced all-cause mortality and death due to bleeding across baseline risk strata	TXA is safe and useful for trauma patients
Brown et al. [19]	Pennsylvania	927 patients	18-90 years, trauma patients	RCT	1 g dose TXA diluted in saline, infused over 10 minutes	Helicopter-transported trauma patients	30-day mortality is lower in the TXA group if administered within one hour of injury	Prehospital TXA did not increase thrombotic complications or adverse events
Mahmood et al. [20]	Multinational	1767 patients	Patients with isolated head injury	RCT	Loading dose 1 g over 10 min, then 1 g infusion over eight hours	Intraparenchymal and intracranial hemorrhage	No evidence that TXA prevents hemorrhage expansion	TXA is not useful for intraparenchymal hemorrhage
Li et al. [21]	Multinational	476 patients	18 to 89 years, trauma patients	RCT	1 g bolus over 10 minutes followed by 1 g over eight hours	Blunt injuries due to vehicle accidents	Early prehospital TXA significantly reduced 30-day mortality	TXA is useful in reducing mortality
Jachetti et al. [22]	Haiti	116 patients	18-65 years, trauma patients	RCT	Loading 1 g of TXA in 10 min, followed by 1 g after three hours	Blunt or penetrating trauma, SATS score ≥ 7	70% less chance of death during hospitalization post Massive Hemorrhage Protocol introduction	No adverse effects; confirms TXA's safety profile
CRASH Trial Collaborators [23]	Multinational	20,211 patients	Adult trauma patients with significant bleeding risk	RCT	Loading dose of 1 g over 10 min then 1 g infusion over eight hours	Various, including bleeding and head injury	Significant reduction in all-cause mortality and death due to bleeding	TXA is effective in reducing mortality in trauma patients
Roberts et al. [24]	London, UK	20,211 patients	Adult trauma patients	RCT	Loading dose 1 g over 10 minutes then 1 g over eight hours	Significant bleeding within eight hours of injury	Significant reduction in all-cause mortality at 28 days	Early treatment (≤1 hour from injury) significantly reduced death due to bleeding
Dewan et al. [25]	London, UK	12,737 patients	Adults with traumatic brain injury	RCT	Loading 1 g dose followed by a 1 g maintenance dose over eight hours	Brain injury within eight hours, with intracranial bleeding	TXA is safe in TBI patients; treatment within three hours reduces head injury-related death	Early treatment is more effective in mild/moderate head injury
The PATCH and ANZIC collaborators [26]	Australia, New Zealand, Germany	1310 patients	Adults, trauma patients	RCT	Loading 1 g before hospital, then 1 g infusion over eight hours	Major trauma with suspected coagulopathy	No difference in survival with a favorable functional outcome at six months	TXA shows favorable outcomes in the functional state

**Figure 2:**
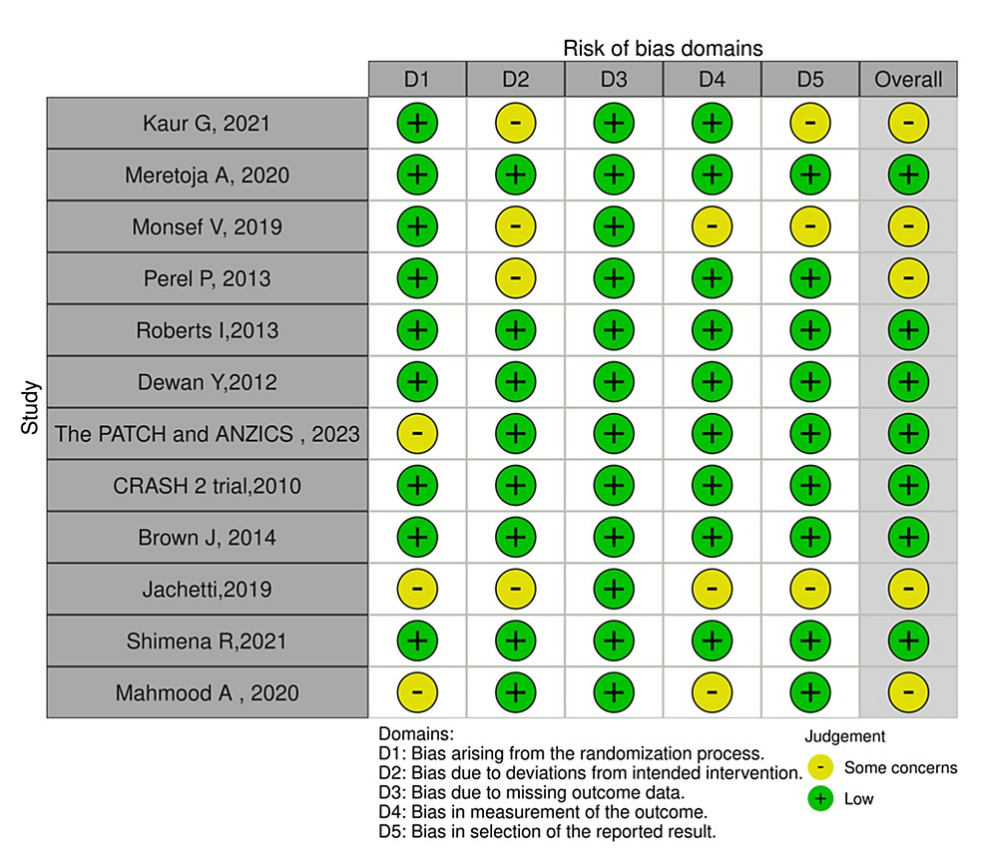
Assessment of risk of bias of included studies Traffic lighting plot assessing the risk of bias of each article.

Based on the analysis, nine of the articles had a low risk of bias, whereas three had some concerns about bias. There were no articles with a high risk of bias across the five domains assessed. Figure 2 below portrays the diagrammatic representation of the risk of bias in all 12 articles. This shows that we can draw reliable conclusions from the studies that can be generalized to the population.

Discussion

The cumulative analysis of various studies on TXA reveals significant insights. A substantial portion of the research, encompassing 60,338 patients, suggests that TXA effectively reduces mortality compared to its absence. This benefit appears consistent across different types and timings of administration. The most prevalent administration method is an initial 1-gram dose followed by an eight-hour infusion, which has not been associated with significant side effects. Particularly noteworthy are the studies by Kaur et al. (2021) and Monsef Kasmaei et al. (2019), which demonstrate TXA's ability to mitigate postoperative declines in hemoglobin and hematocrit levels at 24, 48, and 72 hours, in comparison to placebo [15,17]. However, the efficacy of TXA in treating head trauma yields divergent outcomes. Among 12 studies, two notable ones - Meretoja et al. (2020) and Mahmood et al. (2021), involving 1867 patients - reported no significant benefit in cases of head trauma [16,20]. These findings highlight the possibility that TXA's effectiveness might vary depending on the specific nature of the trauma. Consequently, further research is imperative to ascertain the definitive benefits of TXA in head trauma scenarios, as in our included studies the results showed that TXA does not modify the mortality, morbidity, or any parameter that could be beneficial for the patient, despite this fact a bigger sample size is required to take decisions on head trauma patients confidently.

The systematic review by Augustinus et al. (2023) included 13 articles encompassing 54,843 patients undergoing hemiarthroplasty surgery and found that 14.1% (7733 patients) of these patients who received perioperative TXA experienced a significant decrease in transfusion rates, improved postoperative hemoglobin levels, reduced hospital stay lengths, and lower 30-day mortality [27]. Similarly, a meta-analysis by Liechti et al. in 2023 involving 1139 mastectomy patients revealed that perioperative intravenous administration of TXA reduced the risk of hematoma and seroma formation [28]. Furthermore, a meta-analysis by Liu et al. in 2022, covering 1497 patients undergoing spine surgery, demonstrated that TXA decreased total and perioperative blood loss, postoperative drainage, hospital stay duration, total blood transfusion volume, and international normalized ratio [29]. This previously reported evidence concurs with our findings that demonstrate that TXA can reduce blood loss and improve hemoglobin and hematocrit outcomes in patients undergoing surgery; TXA is useful in a wide variety of surgery scenarios beyond trauma surgery and also in some other scenarios of bleeding [30]. We have already detected that in the trauma context, the use of TXA is useful for pelvic, blunt, open, and general trauma with a reduction of mortality of the patients. Along these trauma pathologies, the diverse studies included in this systematic review have shown that TXA 1 gram intravenous infusion is enough to cause positive effects in the reduction of mortality of these kinds of patients, and the previously mentioned systematic reviews and meta-analyses corroborate with our results, giving external validity to our current findings.

In terms of dosage, our systematic review found several doses being the most common loading of 1 gram and a second dose of 1 gram eight hours after the first one. A previous systematic review by Masouros et al. concluded no significant difference in total blood loss reduction between single and multiple doses of TXA. Moreover, fewer complications were observed in patients who received a single dose of ≤15 mg/kg [31]. This finding is corroborated by research from Qin et al. in 2022, which compared 740 patients receiving high and low doses of intravenous TXA and found no significant differences in terms of total blood loss, perioperative hemoglobin and hematocrit levels, operative time, and blood transfusion rates [32]. These results support our observations that different doses can cause a positive impact on the patients and that the dosage does not change the outcome in reducing mortality and blood loss, also we identify no adverse events in the administration of TXA in our studies that encompass the conclusions of previous studies. For this reason, we conclude that giving TXA in different doses depending on the hospital protocol for the patients under trauma is beneficial for the reduction of mortality and blood loss, without any adverse event related to the administration of this drug.

However, this systematic review faced limitations due to restricted access to articles, with only publicly available articles included, which excluded several privately held studies. Additionally, our review focused exclusively on patients undergoing trauma surgery, which encompasses a wide variety of conditions. Therefore, our findings may not be representative of all trauma pathologies and should be cautiously extrapolated to other trauma-related scenarios. We recommend further research in specific situations such as head trauma to assess effectively the use of TXA in this specific population. Additionally, the long-term events are not reported in the included studies, which is a great limitation for the recommendation of TXA. We recommend that future research focusing on the use of TXA in trauma patients evaluate the long-term events of this medication.

## Conclusions

The extensive TXA analysis across a range of studies robustly supports its efficacy in reducing mortality and enhancing postoperative outcomes, especially in diverse surgical environments. The consistent positive findings, particularly the reduction in transfusion rates, improved hemoglobin levels, and decreased risk of complications, emphasize the significant role of TXA as a versatile therapeutic option for clinicians. Notably, the effectiveness of an initial 1-gram dose followed by an eight-hour infusion and results indicating similar outcomes with a single 15 mg/kg dose have practical implications for clinical practice. These findings suggest that a standardized and simplified TXA dosing regimen can lead to favorable outcomes while minimizing adverse event risks. Nevertheless, the varied results in cases of head trauma underscore the necessity for a tailored approach to treatment, depending on the specific medical condition. Future research should focus on identifying the factors that influence the efficacy of TXA in treating head trauma. This would provide valuable insights, helping clinicians make more informed decisions for this particular group of patients.

Furthermore, upcoming studies should aim to overcome the limitations observed in the current systematic review, such as the limited access to specific articles and the concentration on trauma patients. In-depth research encompassing a broader spectrum of surgical situations and a more diverse patient demographic will lead to a deeper understanding of TXA's effectiveness. This, in turn, will guide clinicians in its optimal use across varied clinical contexts. As TXA continues to show its beneficial impact, further refining its application through dedicated research will enhance its practical utility in patient care. This evolution of TXA's use will not only improve patient outcomes but also reinforce its status in the realm of clinical practice.
